# Patients with *CDH23* mutations and the 1555A>G mitochondrial mutation are good candidates for electric acoustic stimulation (EAS)

**DOI:** 10.3109/00016489.2011.649493

**Published:** 2012-03-25

**Authors:** Shin-Ichi Usami, Maiko Miyagawa, Shin-Ya Nishio, Hideaki Moteki, Yutaka Takumi, Mika Suzuki, Yoko Kitano, Satoshi Iwasaki

**Affiliations:** 1 Department of Otorhinolaryngology, Shinshu University School of Medicine, Matsumoto, Japan; 2 Department of Hearing Implant Sciences, Shinshu University School of Medicine, Matsumoto, Japan; 3 School of Health Sciences, Tokai University, Isehara, Japan

**Keywords:** Residual hearing, hearing preservation, gene, mitochondria, 12S rRNA

## Abstract

*Conclusions: CDH23* mutations and the 1555A>G mitochondrial mutation were identified among our series of electric acoustic stimulation (EAS) patients, confirming that these genes were important in hearing loss with involvement of high frequency. Successful hearing preservation as well as good outcomes from EAS indicated that patients with this combination of mutations are good candidates for EAS. *Objectives:* Screening for gene mutations that possibly cause hearing loss involving high frequency was performed to identify the responsible genes in patients with EAS. In addition to a review of the genetic background of the patients with residual hearing loss, the benefit of EAS for patients with particular gene mutations was evaluated. *Methods:* Eighteen patients (15 late-onset, 3 early-onset) with residual hearing who had received EAS were included in this study. Genetic analysis was performed to identify *GJB2, CDH23, SLC26A4*, and the 1555 mitochondrial mutations. *Results:* Three early-onset patients had *CDH23* mutations. One late-onset patient had the 1555 A>G mitochondrial mutation.

## Introduction

Hearing loss in the majority of patients with residual hearing at lower frequencies is more or less progressive, although the speed of progression, i.e. rapid or rather stable, may be dependent on the etiology. An unresolved issue is the prediction of progressiveness based on the etiology of individual hearing loss. We have recently reported at least four genes that are responsible for the candidates for electric acoustic stimulation (EAS), and therefore there is not a single etiology but rather a great genetic heterogeneity involved in this particular type of hearing loss [1]. In this study, screening for mutations of four genes (*GJB2, CDH23, SLC26A4*, and the 1555 mitochondrial mutations), which possibly cause high frequency hearing loss, was performed to identify the responsible genes for 18 patients with EAS.

## Material and methods

Eighteen patients (8 males and 10 females, aged 1–68 years) were included in this study. Clinical features of the subjects are summarized in Table I. As regards onset of hearing loss, 15 patients were late-onset (10–50 years old) and 3 patients were early-onset (most probably congenital). Anamnestic evaluation and/or serial audiogram indicated that all of the patients had progressive sensorineural hearing loss. No patients had any anomalies such as enlarged vestibular aqueduct. All patients had some residual hearing in the lower frequencies, and therefore received EAS. The round window approach was applied for all thepatients, and intraoperative and postoperative intravenous administration of dexamethasone was used as described in a previous report [2]. For genetic analysis, direct sequencing for *GJB2, SLC26A4, CDH23*, and the 1555 mitochondrial mutation was performed. Detailed methods are described elsewhere [3–6].

**Table 1:** Clinical features of subjects in study.

Case no.	Gender	Age (EAS)	Onset (age)	Progressiveness	Inheritance mode	Responsible gene	Implant	Insertion depth (mm)
1	F	59	Late (43)	+	Sporadic	N/I	PULSAR FLEXeas	24
2	F	71	Late (30)	+	AD	N/I	PULSAR FLEXeas	24
3	F	45	Late (25–30)	+	Sporadic	N/I	PULSAR FLEXeas	24
4	F	38	Late (34)	+	Sporadic	N/I	PULSAR FLEXeas	24
5	F	46	Late (30)	+	AD	N/I	PULSAR FLEXeas	24
6	M	29	Late (10)	+	AD	N/I	PULSAR FLEXeas	24
7	M	39	Late (20)	+	AD	N/I	PULSAR FLEXeas	24
8	F	35	Late (25)	+	Sporadic	N/I	PULSAR FLEXeas	24
9	M	52	Late (25)	+	Mitochondrial	*Mit. 1555A>G*	PULSAR FLEXeas	24
10	F	51	Late (30)	+	AD	N/I	PULSAR FLEXeas	24
11	M	39	Late (6)	+	Sporadic	N/I	PULSAR FLEXeas	24
12	F	45	Late (25)	+	Sporadic	N/I	PULSAR FLEXeas	24
13	F	38	Late (10)	+	AR	N/I	PULSAR FLEXeas	24
14	F	60	Late (40)	+	AD	N/I	Combi 40+ standard	31.5
15	M	68	Late (50)	+	Sporadic	N/I	PULSAR FLEXsoft	31.5
16	M	12	Early (3)*	+	AR	*CDH23*	PULSAR FLEXsoft	31.5
17	M	12	Early (1 year 8 months)*	+	AR	*CDH23*	PULSAR FLEXsoft	31.5
18	M	1	Early (0)†	NA	Sporadic	*CDH23*	PULSAR FLEXsoft	31.5

N/I, not identified within four genes.

* Most probably congenital.

† Newborn hearing screening.

## Results

**Figure 1:**
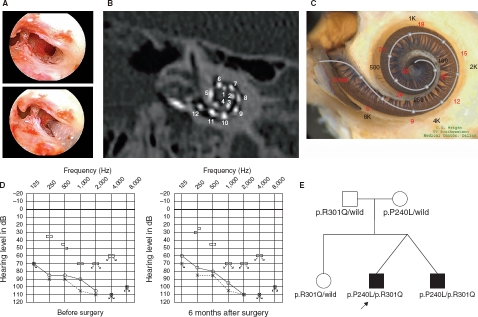
Case no. 16. (A) Endoscopic view of round window insertion, (B) montage CT image, (C) imaging with putative location of electrode and the referential tonotoic map, (D) preoperative and postoperative audiograms. The image of human cochlea neural tissues stained by osmium tetroxide used in Figures 1,2,3,4 was kindly provided by Dr C.G. Wright, USWT, Dallas, USA (red, mm from round window; black, corresponding frequency). (E) Pedigree and the mutations found in the CDH23 gene.

**Figure 2:**
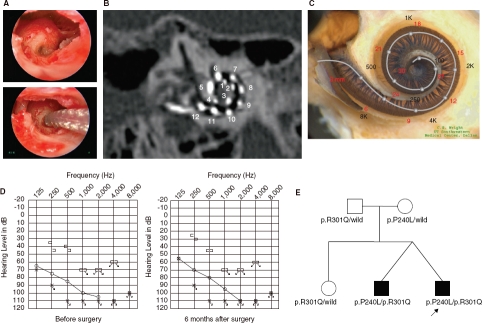
Case no. 17. (A) Endoscopic view of round window insertion, (B) montage CT image, (C) imaging with putative location of electrode and the referential tonotoic map, (D) preoperative and postoperative audiograms. (E) Pedigree and the mutations found in the *CDH23* gene.

**Figure 3:**
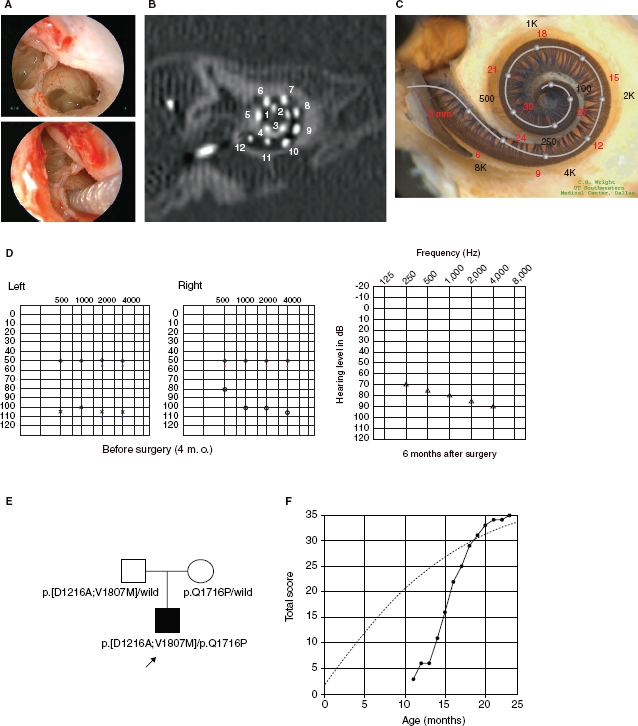
Case no. 18. (A) Endoscopic view of round window insertion, (B) montage CT image, (C) imaging with putative location of electrode and the referential tonotoic map, (D) preoperative ASSR findings (blue, left; red, right) and postoperative COR audiogram finding. (E) Pedigree and the mutations found in the *CDH23* gene. (F) Auditory behavioral development assessed by LittlEARS® Auditory Questionnaire. The development curve shows rapid improvement in auditory behavior reaching the curve of normally developed children.

**Figure 4:**
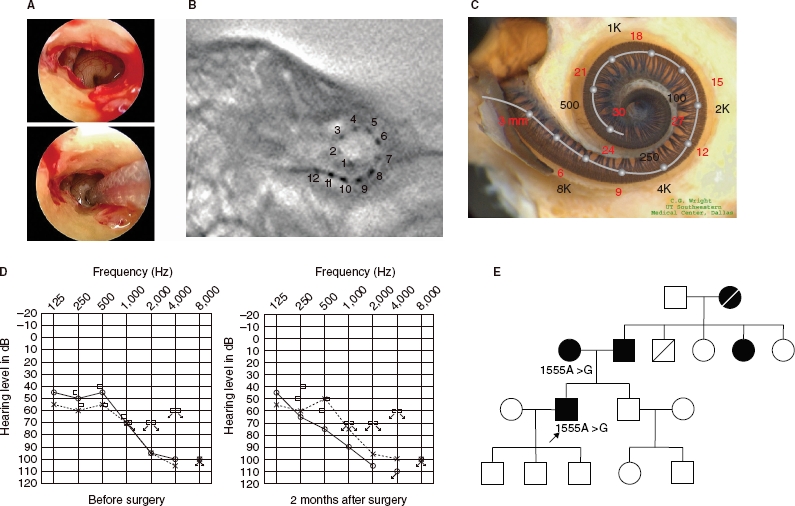
Case no. 9. (A) Endoscopic view of round window insertion, (B) postoperative X-ray finding, (C) imaging with putative location of electrode and the referential tonotoic map, (D) preoperative and postoperative audiograms. (E) Pedigree and the subjects with the mitochondrial 1555 mutations.

All three early-onset patients had *CDH23* mutations (case nos 16, 17, and 18; Figures 1,2,3). One post-lingual patient had the 1555 A>G mitochondrial mutation (case no. 9; Figure 4). Hearing in the low frequencies after cochlear implantation was well preserved in all 18 cases including these 4 cases.

### Case nos 16 and 17 (Figures 1 and 2)

The patients were 12-year-old twins, had the same mutations in the *CDH23* gene, and showed similar audiograms and a slowly progressive nature confirmed by serial audiograms. Both had some residual hearing in the lower frequencies and used hearing aids, but due to the progression of their hearing loss, they received cochlear implants (Nucleus CI24M device, with complete insertion of a straight array through cochleostomy) for the left ear at the age of 5 (no. 16) and 6 (no. 17). In one of the twins (no. 16) residual hearing was successfully preserved

(Figure 1D), but the other (no. 17) lost his air-conduction thresholds after cochlear implantation even though the bone-conduction threshold remained stable (Figure 2D). Their audiological performance was good with the cochlear implantation (electric stimulation only). They wanted to have cochlear implants on the other sides, considering their residual hearing and the progressive nature of the hearing loss, and we decided to use a longer atraumatic electrode (MEDEL PULSAR CI100/FLEXsoft electrode) to cover the low frequencies (Figure 1A, B, C; Figure 2A, B, C). Hearing was well preserved 6 months postoperatively (Figures 1D and 2D). Both had compound heterozygous mutations (p.P240L/p.R301Q), and their parents were found to be carriers for these mutations (Figure 2E). After identification of the *CDH23* mutations, they were referred for ophthalmologic examination including electroretinography (ERG) and visual field analysis. Both had normal ERG response and no visual field deficits, confirming the nonsyndromic phenotype (DFNB12). Furthermore, they did not have any ves-tibular problems and showed normal responses in caloric testing. Their hearing thresholds improved to 30 dB and 35 dB (nos 16 and 17, respectively) (average for all frequencies from 125 to 8000 Hz) 1 year after cochlear implantation. Their word recognition scores in quiet improved from 64% to 76% (no. 16) and from 60% to 76% (no. 17) at 1 year postoperatively.

### Case no. 18 (Figure 3)

This case was a 1-year-old boy with the *CDH23* mutations. Auditory steady-state response (ASSR) evaluated at the age of 4 and 7 months showed some residual hearing at 500 Hz in the right ear (Figure 3D). He first received a left cochlear implant (MEDEL PULSAR CI100/standard electrode) at the age of 9 months. The parents wanted him to use a cochlear implant on the right side as well, and we decided to use a more atraumatic electrode (MEDEL PULSAR CI100/FLEXsoft electrode) because of the possible residual hearing in the low frequencies (Figure 3A, B, C). The second cochlear implant surgery was performed at the age of 12 months. Residual hearing measured by conditioned orientation reflex (COR) audiometry [7] was well preserved 1 year after cochlear implantation (Figure 3D). This patient had compound heterozygous mutations (p.[D1216A; V1807M]/p.Q1716P) and the parents were found to be carriers for these mutations (Figure 3E). Although the patient was too young to undergo ophthalmologic examination, he did not have any problems in vision or any vestibular problems, and there is no indicative evidence for Usher syndrome at this time.

In this very young case, auditory behavioral development was assessed by using the LittlEARS® Auditory Questionnaire, which has been designed for children under the age of 2 years [8,9]. The development curve showed a rapid increase in auditory behavior and reached the score seen in normally developed children (c 3F).

### Case no. 9 (Figure 4)

This case was a 52-year-old male with the 1555A>G mitochondrial mutation. He noticed hearing loss around age 38 and used hearing aids, but his hearing loss was slowly progressive as evaluated by serial audio-grams. Due to residual hearing in the lower frequencies, an atraumatic electrode (MEDEL PULSAR CI100/FLEXeas electrode) was chosen (Figure 4A, B, C). Residual hearing was well preserved at 2 months post-operatively (Figure 4D). His parents had hearing loss, and the pedigree was consistent with mitochondrial inheritance (as well as autosomal dominant inheritance) (Figure 4E). Genetic screening detected the 1555 mitochondrial mutation in the patient and his mother. He had no history of exposure to amino-glycoside antibiotics. No vestibular symptoms were noted, and no abnormal findings were seen in vestibular testing including caloric response and vestibular evoked myogenic potential (VEMP). His hearing threshold improved to 30 dB (average for all frequencies from 125 to 8000 Hz) 2 months after cochlear implantation. Due to an insufficient follow-up period, his speech recognition score has not yet been evaluated.

## Discussion

As predicted from our previous study [1] using patients who fulfilled the criteria for EAS, the *CDH23* mutations and the 1555A>G mitochondrial mutation were in fact found among our series of EAS patients.

Our previous study indicated that the *CDH23* mutations were frequently found in patients with recessive inheritance and the presence of residual hearing is one particular phenotypic feature of the patients with *CDH23* mutations [5], and actually all of the early-onset patients had the mutations in this gene.

The *CDH23* gene encodes cadherin 23, a protein thought to be a molecule that forms the lateral links between the stereocilia of hair cells [10]. One remarkable result in this study is that function of the lateral links remained stable even after deep insertion of the electrode of the cochlear implant. Such functional preservation enabled hearing preservation even in the presence of an electrode covering the corresponding frequency region.

As suggested by genotype–phenotype correlation study, USH1D, which has a more severe phenotype including severe to profound hearing loss, vestibular dysfunction, and retinitis pigmentosa, is usually associated with nonsense, splicing-site, and frameshift mutations. In contrast, DFNB12, which has a milder phenotype, is associated with missense mutations [11,12]. The mutations found in the present three cases (we previously reported case nos 16 and 17 as family no. 3 [5]) are consistent with the general genotype–phenotype correlation rule.

In Usher type I patients, known to have the same etiology, improvement in sound detection as well as speech perception was seen in all patients, especially younger ones [13]. The present study clearly indicates that patients with the *CDH23* mutations are good candidates for EAS. The previous report together with the present cases indicates that progressiveness of hearing loss is a characteristic feature of the patients with this mutation [5,12]. Therefore, deep insertion with longer electrodes is recommended to prevent future deterioration. Successful hearing preservation and prediction of future hearing level by genetic diagnosis may facilitate decision making for early intervention.

It is interesting that *GJB2*, the most prevalent causative gene among the prelingual patients, was not found in the present series of patients. This is probably due to their more or less flat audiograms [1] and therefore they may be good candidates for conventional cochlear implantation.

In very young children, pure tone audiograms are not available. Acoustic brainstem response (ABR) is usually used to evaluate their hearing, but it is difficult to measure residual hearing in the low frequencies. Recently, acoustic steady-state response (ASSR) has been clinically available to measure hearing levels of 500 Hz or 250 Hz, but sometimes the low frequency part is not reliable or convincing [14]. In addition to such hearing testing, genetic testing is useful to predict the residual hearing at low frequencies. Especially for cases with *CDH23* mutations, predicted audiograms can be obtained for the very young patients. Based on this concept, together with consideration of their expected long life (which includes a risk of progression), we chose a longer atraumatic electrode (MEDEL PULSAR CI100/FLEXsoft electrode) for three patients with *CDH23* mutations.

It is known that patients with the 1555A>G mito-chondrial mutation are susceptible to aminoglycoside antibiotics [15]. The 1555A>G mutation is one of the most important mutations among the hearing loss population in Japan, and approximately 3% of patients with sensorineural hearing loss possess this mutation [16]. Their hearing loss is known to be slowly progressive [6,17]. This mutation is an important cause in the post-lingual cochlear implant patients, found in 10% of them [16]. It has been reported that a patient with cochlear implantation showed excellent auditory performance [18], indicating that cochlear implantation is a valuable choice of therapy for patients with profound hearing loss caused by this mutation. This mutation was also found in patients without any aminoglycoside exposure and their hearing loss was usually milder than those with aminoglycoside exposure [19]. Environmental causative factors other than aminoglycoside antibiotics – such as noise or mechanical stress – have been speculated, although not confirmed. The present study provided an important clinical experience that EAS could be safely performed even if the patients have this mutation and therefore possible association of susceptibility for any mechanical stress.

For outcome of EAS, together with successful hearing preservation, all four patients obtained 25–35 dB in average hearing threshold after implantation. Since EAS was implanted as a second cochlear implant for three cases with *CDH23* mutations, it is difficult to evaluate the independent benefit of EAS. However, improvement of word recognition scores after EAS was observed in case nos 16 and 17, indicating that additive benefit was clearly obtained even after a rather long period following the first implants (at 7 years and 6 years, respectively). For case no. 18, although it is also difficult to evaluate the independent benefit of EAS because of the very young age, the auditory behavioral development as assessed by the LittlEARS® Auditory Questionnaire was significantly improved after two consecutive implantations. Since the *CDH23* mutation will be potentially found in rather young candidates, this genetic marker could be available for the existence of residual hearing. For those patients, it is strongly suggested that the surgeon keep in mind the option of performing atraumatic surgery.

In the present series, there are many families with autosomal dominant hearing loss (6 of 18), suggesting that many other genes responsible for dominant hearing loss may be involved. It is also important to note that all of the patients showed progressive hearing loss. We are currently searching for the responsible genes for the patients with high frequency hearing loss.

In conclusion, the *CDH23* mutations and the 1555A>G mitochondrial mutation were identified among our series of EAS patients, confirming that these genes were important in high frequency hearing loss. Successful hearing preservation in these patients as well as good outcomes of EAS indicated that those with these mutations are good candidates for EAS. The present study indicates that genetic testing provides useful information regarding residual hearing and consequent therapeutic options.
